# The Current State of Knowledge on the Clinical and Methodological Aspects of Extracorporeal Shock Waves Therapy in the Management of Post-Stroke Spasticity—Overview of 20 Years of Experiences

**DOI:** 10.3390/jcm10020261

**Published:** 2021-01-12

**Authors:** Józef Opara, Jakub Taradaj, Karolina Walewicz, Joanna Rosińczuk, Robert Dymarek

**Affiliations:** 1 Department of Clinical Rehabilitation, Academy of Physical Education in Katowice, 40-065 Katowice, Poland; jozefopara@wp.pl; 2 Institute of Physiotherapy and Health Sciences, Academy of Physical Education in Katowice, 40-065 Katowice, Poland; j.taradaj@awf.katowice.pl; 3 College of Rehabilitation Sciences, University of Manitoba, Winnipeg, MB R106-771, Canada; 4 Department of Rehabilitation, TOMMED Medical Center in Katowice, 40-662 Katowice, Poland; 5 Institute of Health Sciences, University of Opole, 45-060 Opole, Poland; karolina.w101@wp.pl; 6 Department of Nervous System Diseases, Faculty of Health Sciences, Wroclaw Medical University, 51-618 Wroclaw, Poland; joanna.rosinczuk@umed.wroc.pl

**Keywords:** stroke, muscle spasticity, shock wave therapy, physiotherapy, neurorehabilitation, state of art, narrative review

## Abstract

In many patients after stroke, spasticity develops over time, resulting in a decrease in the patient’s independence, pain, worsening mood, and, consequently, lower quality of life. In the last ten years, a rich arsenal of physical agents to reduce muscle tone such as extracorporeal shock therapy (ESWT) wave has come through. The aim of this narrative review article is to present the current state of knowledge on the use of ESWT as a supplement to the comprehensive rehabilitation of people after stroke suffering from spasticity. The PubMed and PEDro databases were searched for papers published in English from January 2000 to December 2020, 22 of which met inclusion criteria for clinical studies related to post-stroke spasticity management with ESWT. A total of 22 studies including 468 post-stroke patients—11 reports with the upper limb (267 patients) and 10 reports within the lower limb (201 patients), as well as one report including both upper and lower limb. We focused our attention on clinical and methodological aspects. Therefore, we performed the assessment of enrolled studies in terms of methodological quality using the PEDro and level of evidence using the National Institute for Health and Clinical Excellence (NICE) guidelines. Furthermore, we indicated implications for clinical practice in using ESWT for post-stroke spasticity management. Moreover, we discussed a suggestion for future research directions. In conclusion, an ESWT effectively reduces muscle tone in people with spastic limb after stroke. Further, ESWT is safe and free of undesirable side effects. The mechanism of action of ESWT on muscles affected by spasticity is still unknown. To date, no standard parameters of ESWT in post-stroke spasticity regarding intensity, frequency, location, and the number of sessions has been established. Further research, meeting the highest standards, is needed to establish uniform muscle stimulation parameters using ESWT.

## 1. Introduction

According to a recent paper by Bensmail et al. [1] post-stroke spasticity is estimated to affect up to 43% of stroke survivors and can be seen already in the first week after stroke onset. Spasticity is more common in the upper than lower extremity and is proportional to the severity of upper-limb impairment—the prevalence of post-stroke spasticity increases during the first year [2]. Based on a systematic review of the literature Schinwelski and Sławek [2] found that spasticity within 12 months after stroke develops in 14–44% of patients.

In the Urban et al. [3] study, from 211 patients with ischemic stroke spasticity developed in 41.6% within six months. According to Wissel et al. [4], spasticity manifests itself in 4 to 27% of patients in the first four weeks after stroke onset, in 19 to 26.7% in the second and third month after the disease and in 17 to 42.6% in the phase chronic (>3 months after onset)—the discrepancy of these data results from the difference in the results of observations of different authors. Zorowitz et al. [5] reported that spasticity would develop in about 20–40% of people who survived stroke. According to Yelnik et al. [6] spasticity can reach even 40–70% in the chronic phase of stroke. This brief overview shows how important it is to follow up patients with increased risk of developing spasticity to start adequate treatment and prevent the negative consequences of spasticity.

Since 2005, commonly used is the definition made by experts under the leadership of Pandyan [7]: (Spasticity is) “a disordered sensory-motor control, resulting from an upper motoneuron lesion presenting as intermittent or sustained involuntary activation of muscles”. Severe spasticity may hamper rehabilitation, reduce the functionality, limit the patient’s autonomy, and lead to contractures, pain, and weakness. Spasticity may impair the ability to perform activities of daily living (ADL), household tasks, self-care, may decrease mobility, can cause such symptoms like pain, sleep disturbances, mood changes, depression, anxiety, and may also reduce patient’s quality of life and cause a negative impact on family and caregiver’s relationship [5,8].

### 1.1. Development of Post-Stroke Spasticity

Spasticity, a neurological impairment, is a common but inevitable consequence of an upper motor neuron (UMN) syndrome. It is one of many sensory-motor signs and symptoms that may be present following an UMN lesion [7]. Lesions involving both the pyramidal and parapyramidal pathways cause increased excitability of alpha-neurons in skeletal muscle.

It is hard to predict the development of spasticity after stroke. Kong et al. [9] evaluated the occurrence and temporal evolution of UL spasticity in 163 patients with a first-ever ischemic stroke (NIHSS 8.5 ± 5.2, UEMI 35.8 ± 30.8, mean age 63.8 ± 10.7 years) admitted to a rehabilitation unit. Spasticity of UL occurred in 33% of patients at three months after stroke. The development of spasticity at later stages of the stroke was infrequent, occurring in only 17%. In patients with mild spasticity (Ashworth Scale score 1) at three months after stroke, the worsening of spasticity occurred in only one patient.

On the other hand, almost half of the patients with moderate spasticity (Ashworth Scale score 2) at three months progressed to severe spasticity (Ashworth Scale score 3). Reduced UL activity was the most important correlate of “moderate to severe spasticity” (Ashworth Scale score ≥ 2) (*p* < 0.001), and poor UL strength on admission to rehabilitation, the most important predictor of “moderate to severe spasticity” (*p* < 0.001). In conclusion: Selective monitoring to detect severe spasticity is recommended for patients with an Ashworth Scale score of two or greater at three months after stroke, and in patients with severe UL weakness on admission to rehabilitation [9].

Lundström et al. [10] stated that the prevalence of any spasticity 12 months after first-ever stroke was 17% and of disabling spasticity 4%. Patients with DS scored significantly worse than those with no disabling spasticity (DS) on the Modified Rankin Scale (MRS) (*p* = 0.009) and the BI (*p* = 0.005). Disabling spasticity was more frequent in the upper extremity. It positively correlated with other indices of motor impairment and inversely with age. There was an independent effect of severe upper extremity paresis and age below 65 years. Opheim et al. [11] evaluated the upper-limb (UL) spasticity during the first year in 117 patients after the first-ever stroke. Spasticity was present in 25% of the patients at day 3 and 46% at 12 months. In most patients with spasticity, the severity increased during the first year after stroke. Spasticity appeared first in the elbow flexors and later in the elbow extensors and the wrist flexors. The patients with spasticity had a significantly worse sensorimotor function, and more pain reduced joint ROM, and reduced sensibility.

Sommerfeld and et al. [12] assessed the occurrence of spasticity after first-ever stroke (SSS 0–18, 35 men, the mean age of 78 ± 9.5 years) in 95 patients and its association with motor impairments and activity limitations. Spasticity was present in 19% of the patients investigated three months after stroke. Severe disabilities were seen in almost the same number pf patients with or without spasticity.

Wissel et al. [13] based on a literature survey, stated in 2015 that post-stroke spasticity could be a key reason for patients failing to meet physiotherapy goals.

### 1.2. Physical Therapy for Post-Stroke Spasticity

In the last decades, various non-pharmacological interventions have been described in managing spasticity in various neurological conditions. They are used as an adjunct therapy to conventional routine care (pharmacological and rehabilitation) [14].

There is a wide range of well-evidenced neurorehabilitation methods used along with pharmacological agents for management of spastic muscle and improvement of motor recovery after stroke. A common neurophysiological concept is neurodevelopmental treatment by Bobath (NDT) and proprioceptive neuromuscular facilitation by Kabat and Knot (PNF) [15,16,17].

We can include such rehabilitation exercise methods, such as constraint-induced movement therapy (CIMT), sensorimotor movement training (SMT), task-related training (TRT), robot-assisted training (RAT), whole body vibration training (WBVT), mirror visual feedback training (MVFT), electromyographic biofeedback training (EMG-BTF), and virtual reality-based training (VRBT). Moreover, there physical therapy agents can be also used, such as neuromuscular electrical stimulation (NMES), electromyography neuromuscular electrical stimulation (EMG-NMES), transcutaneous electrical nerve stimulation (TENS), functional electrical stimulation (FES), therapeutic ultrasound (TU), as well as cryotherapy (cold water, ice packs, and evaporative sprays), thermotherapy (hot water, sauna, and infrared heat), hydrotherapy, and acupuncture [15,18,19].

Neuromodulative methods, such as repetitive transcranial magnetic stimulation (rTMS) or transcranial direct current stimulation (tDCS), are quite promising to be useful in reducing spasticity after a stroke, but still need to be confirmed by larger and multicenter randomized controlled trials to provide evidence of effectiveness under different neurological conditions [20,21,22].

Despite the available range of non-pharmacological interventions for spasticity, there is a lack of high-quality evidence for many modalities. Khan et al. [14] included 18 systematic reviews to evaluate the evidence for a range of non-pharmacological interventions currently used in managing spasticity in various neurological conditions. There is “moderate” evidence for NMES and acupuncture as an adjunct therapy to conventional routine care (pharmacological and rehabilitation) in persons following stroke. “Low” quality evidence for rehabilitation programs targeting spasticity (such as CIMT, stretching, dynamic elbow-splinting, extracorporeal shock therapy (ESWT) in brain injury; tDCS in stroke; rTMS and TENS for other neurological conditions; and physical activity programs, WBVT, and stretching for other neurological condition. For other interventions, evidence was inconclusive.

Over the last 20 years, especially during the last 5 years due to the intensification of research in this area, it has become proven that the ESWT procedure is a safe and effective alternative method to reduce muscle spasticity in post-stroke stroke patients (but also in spasticity related to cerebral palsy, multiple sclerosis, spinal cord injuries, and brain injuries) as a valuable adjuvant modality to standard treatment and rehabilitation [23].

All experts agree that ESWT could not be the only method of treatment spasticity. A short wave can only support comprehensive rehabilitation. Reviewing the multidisciplinary rehabilitation following botulinum toxin and other focal intramuscular treatment for post-stroke spasticity Demetrios et al. [24] stated that the optimal types (modalities, therapy approaches, and settings) and intensities of therapy for improving activity (active and passive function) in adults with post-stroke spasticity, in the short and longer-term, are unclear. Further research is required to build evidence in this area.

### 1.3. Shock Waves for Post-Stroke Spasticity

According to Wang et al. [25] extracorporeal shock wave therapy (ESWT) is used since 1982 for lithotripsy to treat kidney stones, urinary calculi, and biliary calculi using an acoustic pulse. It is also reported to be used for salivary stones and pancreatic stones. ESWT is a non-invasive treatment that involves creating a series of low energy acoustic wave pulsations that are directly applied to an injury through a person’s skin via a gel medium. There are three types of ESWT: fSWT (focused shock wave therapy), rSWT (radial shock wave therapy), or pSWT (planar shock wave therapy). Recently rSWT in connection with fSWT is mostly used. In the past 15 years, ESWT had emerged as the leading choice in the treatment of many orthopedic disorders, including proximal plantar fasciitis of the heel, lateral epicondylitis of the elbow, calcific tendinitis of the shoulder, and non-union of long bone fracture. Many different parameters of ESWT are used such as energy flux density (EFD): 0.01–0.5 mJ/mm^2^, pressure: 10–100 MPa, number of pulses: 1200–4000, and frequency: 1–12 Hz. This enables the penetration of shock waves from 3 cm up to 12.5 cm depth (theoretically). ESWT is widely used for acute and chronic musculoskeletal disorders including low back pain [26,27].

According to the findings of basic sciences research, the ESWT has been shown to promote the activation of a number of molecular and immunological reactions resulting in improved blood circulation, stimulating angiogenesis and neovascularization reactions as well as activating anti-inflammatory responses. Moreover, strong regenerative properties of ESWT towards increased fibroblast recruitment and reduced tissue apoptosis have been observed [28]. Moreover, it was proven that ESWT increases the activation of the vascular endothelial growth factor (VEGF) and its neuroprotective properties as well as the expression of neurotrophin-3 (NTH-3) which improves the neuroregenerative processes. In addition, the ESWT stimulates neurogenesis by increasing proliferation of neural stem cell (NSC), which can improve the functioning of the nervous system [29]. The mechanisms of ESWT action on spasticity reduction are still under investigated; however, there are a few hypotheses, which attempt to explain these mechanisms. First of all, it is being suggested that ESWT is responsible for inducing nitric oxide (NO) synthesis, which is responsible for the formation of new neuromuscular junctions. Another hypothesis concerns the reduction of excitability of motor neurons by the production of continuous or intermittent pressure on the tendons by the ESWT. It is also suspected that ESWT shows antispastic effects by temporarily disturbing neuromuscular transmission in terms of reducing acetylcholine receptors in neuromuscular junctions [30].

There is also a division according to which there are common mechanisms of biological action for fSWT and rSWT, such as increasing the permeability of cell walls, stimulation of microcirculation (of the blood and lymphatic system), and release of substance P (SP) as a neurotransmitter involved in a multitude of neuronal signaling pathways and responsible for pain modulation. However, there are also mechanisms that only characterize fSWT, such as cavitation, release of nitric oxide (NO) responsible for increased cellular metabolism, neovascularization, angiogenesis, and anti-inflammatory effects, as well as stimulation of growth factors, e.g., fibroblast growth factors (FGF) and transforming growth factor (TGF) [31]. Moreover, the theory of mechanotransduction as a biological pathway explaining a cellular effect should be mentioned in the context of biological mechanisms of ESWT action. Mechanobiology is a quite new branch of biological sciences and the ESWT as the mechanical stimulation (mechanotherapy) has a special place and it is under research interest [31,32].

In view of the above, there is still a justified need to explore a new safe and effective methods improving motor recovery and reducing spasticity after stroke. Further, there is a need for developing a strong clinical guideline based on the existing knowledge from well-designed clinical trials and metanalyses. This is a narrative review showing the current state of knowledge on the clinical and methodological aspects of ESWT in the treatment of post-stroke spasticity can be a valuable summarization of over 20 years of scientific accomplishments and practical experiences in this field.

## 2. Methods

The PubMed and PEDro databases were searched for papers published in English (available in full-text version) using MeSH keywords: “extracorporeal shock wave therapy”, “shock waves”, “stroke”, “muscle spasticity”, and “hemiplegia”. The search covered a period from January 2000 to December 2020. The reference list of obtained articles was also reviewed for additional information. Further, human clinical articles related to ESWT in post-stroke spasticity were subjected to a comprehensive analysis.

The inclusion criteria were (1) reviewed original clinical trials regardless of study design, (2) age of participants over 18 years, (3) patients after ischemic or hemorrhagic stroke regardless of its onset, and (4) the use of ESWT as a method supporting management of spasticity regardless of the primary treatment, pharmacological treatment as well as physiotherapy and rehabilitation.

It should be pointed out that only studies including post-stroke aetiology of muscle spasticity were considered. Duplicated articles with the same data set, studies without sufficient data, and those which did not meet the inclusion criteria were excluded. All articles were reviewed and analyzed by the first author (J.O.). Received results were checked for accuracy by the two co-authors (K.W. and J.R.). Any discrepancies, if any, were resolved through discussion and consensus.

Moreover, the methodological quality of included clinical trials was assessed using the Physiotherapy Evidence Database (PEDro) score (Table A1 (Appendix A)) [33] and the National Institute for Health and Clinical Excellence (NICE) to establish level of evidence (Table A2 (Appendix A)) and level of recommendation (Table A3 (Appendix A)) [34]. Two co-authors (J.T. and R.D.) independently performed the quality assessment on the included clinical trials. In the event of disagreement, the co-authors reviewed the original article to reach a consensus.

## 3. Results

**Figure 1:**
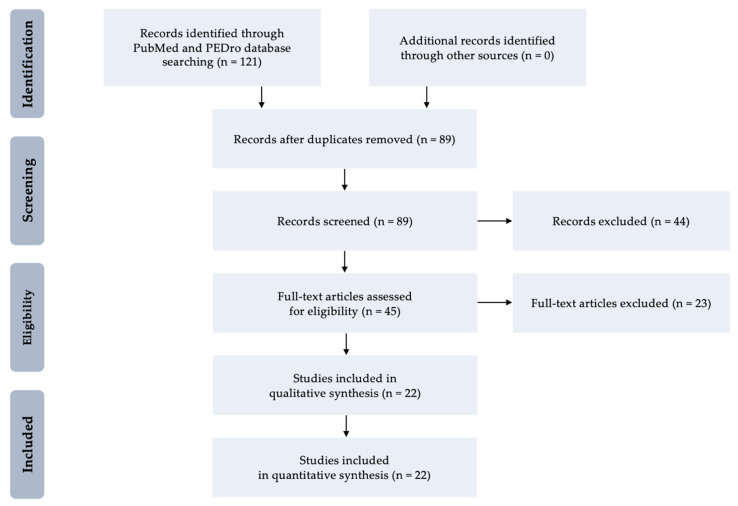
Flow diagram for selection process and identification of studies for inclusion.

At the first stage, 121 clinical trials were identified, 22 of which met criteria for inclusion and at the second stage they underwent a detailed analysis based on qualitative and quantitative syntheses. The enrolled studies were subdivided in terms of the localization of the spasticity into upper or lower limb. A total of 22 studies including 468 post-stroke adults—11 reports with the upper limb (267 patients) [35,36,37,38,39,40,41,42,43,44,45] and 10 reports within the lower limb (201 patients) [46,47,48,49,50,51,52,53,54,55], as well as one report including both upper and lower limbs [56]. Figure 1 presents low diagram for selection process and identification of studies for inclusion in this review.

### 3.1. Shock Waves for Upper Limb Spasticity

In this review, 12 studies including a total group of 267 post-stroke patients with mean age of 59.78 were qualified. The summary of patients’ characteristics and study outcomes post-stroke patients with upper limb spasticity who were treated with ESWT are presented in Table 1.

**Figure 2:**
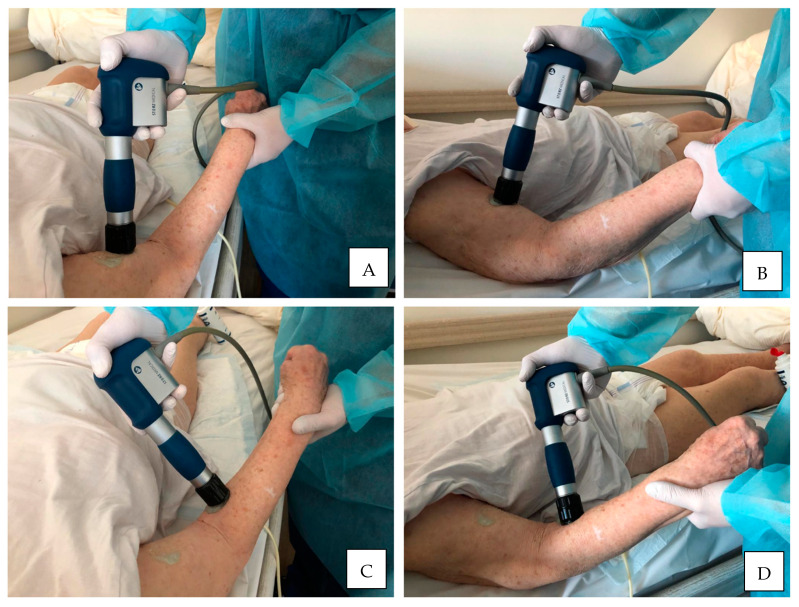
An exemplary methodology of rSWT application for the upper limb muscles. (**A**,**B**), treatment session delivered to the elbow flexors; (**C**,**D**), treatment session delivered to the wrist flexors.

A mean number of ESWT session performed for UL muscles was 3.73 including 2227 pulses delivered with frequency of 6.78 Hz, pressure of 1.91 bars and EFD of 0.09 mJ/mm^2^. The summary of ESWT procedures’ characteristics and other treatments for upper limb spasticity in the course of the study are presented in Table 2 along with an exemplary methodology of rSWT application for the UL muscles in Figure 2.

In terms of methodological quality of analyzed studies using ESWT spastic UL muscles, the mean PEDro score was 5.79 indicating the level B of recommendation accordingly with the NICE guidelines. The results of methodological quality assessment with PEDro score and level of evidence with NICE tool for studies using ESWT in treatment of upper limb spasticity are presented in Table 3.

Manganotti and Amelio (2005) [35] performed the first report on using fSWT in 20 post-stroke patients with severe spasticity in wrist flexor and fingers (11 men, 38 to 76 years old, 15 ischemic, National Institutes of Health Stroke Scale (NIHSS) 10 to 15, at least nine months after onset. They observed significant improvement in the Ashworth scale compared with placebo stimulation at the four-week follow-up visit. At 12 weeks after therapy, 10 of the 20 patients showed a persistent reduction in muscle tone.

Santamato et al. (2013) [36] published the SBOTE study, in which they compared fSWT to electrical stimulation (ES) after BTX-A injections in 32 patients at least six months after stroke (18 women; mean age 63.75 ± 6.43 years). ES was given in UL spasticity for 30 min twice a day for five days starting at 5 Hz; fSWT was given once a day for five days. At study follow-up, patients treated with BTX-A injections and ESWT showed a greater significance and continuous decrease of spasticity in MAS at 15-, 30-, and 90-days post-treatment, spasms frequency, and pain (*p* < 0.05).

Troncati et al. (2013) [37] described a case series of 12 patients with chronic hemiplegia who were treated with two sessions of fSWT. The MAS showed a significant reduction of spasticity and Upper Limb—Fugl–Meyer Assessment (UL-FMA) scores showed improvement in passive range of motion (pROM) immediately after treatment. Persistent effects were observed at 3 and 6 months for MAS and for motricity and pROM subscores of the UL-FMA.

Daliri et al. (2015) [38] in a single-blind clinical trial investigated the effects of a single session rSWT on wrist flexor spasticity in 15 patients after stroke (12 male, mean age 54 years). In results, the MAS scores of spasticity improved, and the improvements were maintained for five weeks.

Dymarek et al. (2016) [39] in their primary open-label clinical trial stated that a single session of rSWT could be an effective alternative treatment for the reduction of UL spasticity as measured in MAS, improvement of resting bioelectrical activity as measured by surface electromyography (sEMG), and could improve trophic conditions of the spastic muscles evaluated with non-invasive and noncontact infrared imaging. They applied rSWT for spastic flexor carpi radialis and flexor carpi ulnaris in 20 chronic stroke patients (13 men, aged 63.15 ± 12.60, nine months till 120 months after onset). The positive results lasted for 24 h.

Dymarek et al. (2016) [40] in their second study with randomized controlled design presented findings among 60 stroke patients who were assigned into active-rSWT group (*n* = 30) and placebo-rSWT group. All patients were analyzed for clinical outcomes using the MAS of the elbow, wrist and finger flexors. Further, surface electromyography (sEMG) and infrared thermography (IRT) were used. All assessments were performed at baseline (T0), immediately after (T1) as well as 1 and 24 h following rSWT finalization (T2 and T3). A single session of rSWT performed in muscle bellies the carpal flexor radials and ulnas using 1500 pulses, EFD of 0.030 mJ/mm^2^, pressure of 1.5 bar, and frequency of 5 Hz. It was observed a statistically significant reduction in the MAS score in comparison to sham rSWT. Significant changes in sEMG activity and temperature distribution in IRT detection was observed. No significant changes were shown in patients after sham-rSWT.

Li et al. (2016) [41] presented the effect of rSWT on the spasticity of the UL in patients with chronic stroke. Sixty patients were divided into three groups: group A received one session of rSWT per week for three consecutive weeks; group B received a single session of rSWT; group C received one session of sham rSWT per week for three consecutive weeks. Compared to the control group, the significant reduction in spasticity of hand and wrist measured in MAS lasted at least 16 and eight weeks in group A and B, respectively. Three sessions of rSWT had a longer-lasting effect than one session.

Kim (2016) [45] performed a placebo-controlled RCT among 34 patients with hemiplegic shoulder who were randomly enrolled into the active rSWT (*n* = 17) and placebo-rSWT (*n* = 17) groups (interventions were administered four times a week for two weeks). The following outcomes have been assessed: VAS and Constant-Murley score (CMS) as well as MAS, FMA, and ROM. VAS was improved at the two-week and four-week follow-up after active rSWT (*p* < 0.05). Moreover, baseline CMS post-intervention and at the two-week follow- up in comparison to placebo group (*p* < 0.05). The remaining outcomes have been improved, however not statistically significant (*p* > 0.05).

Yoon et al. (2017) [56] included 80 patients with spasticity on the elbow flexor and 44 patients on the knee flexor for after chronic stroke a prospective, randomized clinical trial. The patients were at a different time after onset and different ages. They received three fSWT sessions (0.068–0.093 mJ/mm^2^, 1500 shots) one per week at the muscle belly or myotendinous junction or no application (controls). All of them received physiotherapy, and about half received antispastic medication. The MAS and MTS of both the belly and the junction groups showed positive effects from the fSWT on spasticity in the elbow and knee flexors, but the control group did not. The results also tended to improve after each session until the entire intervention was completed. However, there was no significant difference between the belly and junction groups.

Wu et al. (2018) [42] in a randomized noninferiority trial compared rSWT to BTX-A) in the treatment of post-stroke UL spasticity. A total of 42 patients with chronic stroke (28 men; mean age 61.0 ± 10.6 years) have been enrolled. The authors concluded that ESWT is a non-inferior treatment alternative to BTX-A for post-stroke UL spasticity. ESWT and BTX-A caused a similar reduction in the wrist and elbow flexors spasticity; however, ESWT yielded more significant improvement in the wrist and elbow pROM and UL-FMA scores.

Park et al. (2018) [43] in their randomized study enrolled a group of 30 patients after stroke which was divided into ESWT group (*n* = 15) and placebo-ESWT group (*n* = 15). Both ESWT (wrist flexors = 1500 pulses, and plantar interosseous = 3200 pulses, 800 per each muscle; 1.5 bars and 0.03 mJ/mm^2^) and sham-ESWT (only a sound of shocks) interventions were performed (eight sessions, two times per week). Myotonometric measurements were used to for muscle tone and FMA was used to determine motor recovery. Significantly better results were found in active ESWT group than placebo.

Li et al. (2020) [44] performed the only one study in year 2020, which was a randomized, single-blind clinical trial. Post-stroke patients were randomized into control (A, *n* = 25), and two comparative groups where the five sessions of rSWT was applied on agonist (B, *n* = 27) and antagonist muscles (C, *n* = 30). Conventional physical therapy was continued. The assessments were performed using MAS, MTS, VAS, FMA, and swelling scale (SS). It was shown that rSWT is an effective for post-stroke spasticity for both agonist and antagonist muscles as well as it was also beneficial for pain level but had no effect on functional status or swelling of the UL.

### 3.2. Shock Waves for Lower Limb Spasticity

In this review, 11 studies including a total group of 201 post-stroke patients with mean age of 55.38 were qualified. The summary of patients’ characteristics and study outcomes post-stroke patients with lower limb spasticity who were treated with ESWT are presented in Table 4.

**Figure 3:**
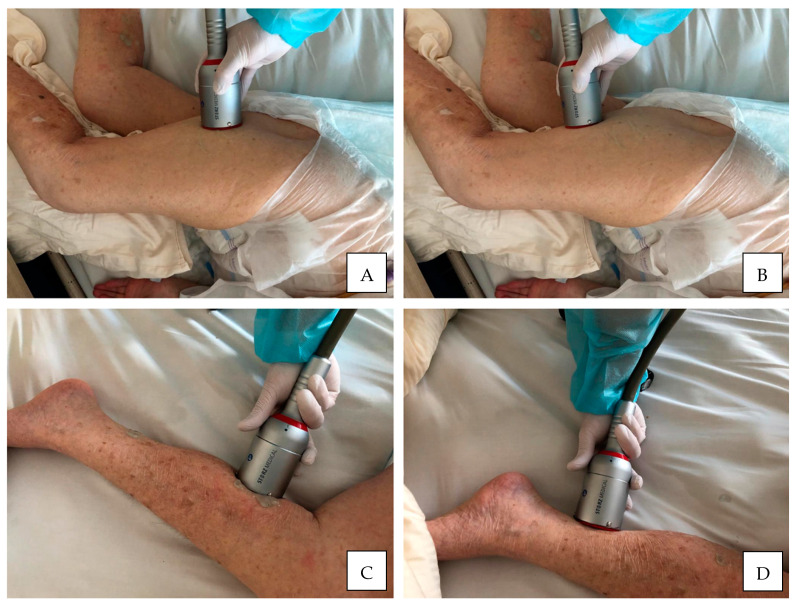
An exemplary methodology of fSWT application for the lower limb muscles. (**A**,**B**), treatment session delivered to the hip extensors; (**C**,**D**), treatment session delivered to the ankle plantar flexors.

A mean number of ESWT session performed for LL muscles was 2.00 including 1750 pulses delivered with frequency of 4.56 Hz, pressure of 1.77 bars and EFD of 0.011 mJ/mm^2^. The summary of ESWT procedures’ characteristics and other treatments for lower limb spasticity in the course of the study are presented in Table 5 along with an exemplary methodology of rSWT application for the LL muscles in Figure 3.

In terms of methodological quality of analyzed studies using ESWT for spastic LL muscles, the mean PEDro score was 4.73 indicating the level C of recommendation accordingly with the NICE guidelines. The results of methodological quality assessment with PEDro score and level of evidence with NICE tool for studies using ESWT in treatment of lower limb spasticity are presented in Table 6.

Sohn et al. (2011) [46] evaluated the electrophysiologic effects of applying one fSWT session to the medial head of the gastrocnemius in 10 hemiplegic stroke patients (six men, mean age of 44.9 ± 11.3 years, two ischemic, 53.4 ± 23.9 month after onset) with ankle plantar flexor spasticity. They stated that spasticity of the ankle plantar flexor was significantly improved in the MAS, with no changes of F wave or H-reflex parameters.

Moon et al. (2013) [47] studied 30 hemiplegic subacute stroke patients (17 males, mean age of 52.6 ± 14.9 years, 16 ischemic, 80.5 ± 46.5 days after onset) with spasticity in the ankle plantar flexor. The fSWT was applied for one session per week, with three sessions at the musculotendinous junction of medial and lateral gastrocnemius muscles. Spasticity in the MAS significantly improved immediately and one week after fSWT. However, these changes were not significant at four weeks after ESWT.

Santamato et al. (2014) [48] published a prospective open-label, the authors examined 23 patients with post-stroke lower limb (LL) spasticity with spastic foot who received one fSWT session on hypertonic plantar-flexor muscles. They stated that fSWT is efficacious for reducing muscle tone and improving passive ankle dorsiflexion motion. The effect was long-lasting in subjects with echo intensity of calf muscles graded I, II, or III but was brief for echo intensity graded IV on the Heckmatt scale [57].

Kim et al. (2015) [49] evaluated the effects of three sessions rSWT, and after the last session, they performed stretching exercises for Achilles tendon and plantar fascia for 30 min/day, five times a week for six months on 10 stroke patients with plantar fasciitis (five men, age 64.10 ± 4.01 years, 17.60 ± 2.36 month after onset). In results: six weeks after therapy, and six months after therapy thickness of the plantar fascia, degree of spasticity, pain, and gait ability has been improved. These changes were significantly more significant at six months after therapy than at six weeks after therapy.

Radinmehr et al. (2017) [50] treated plantar flexor spasticity in 12 patients (seven male, five female) in the age between 42 and 78 years, and 4–60 months after stroke onset, with single rSWT (0.340 mJ/mm^2^, 2000 shots). They noticed the improvement of the MMAS scores for both the gastrocnemius and the soleus muscles, active and passive ROM, passive plantar flexor torque (pPFT), and TUG one hour after rSWT. The rSWT had no significant effects on alpha motoneuron excitability.

Sawan et al. (2017) [51] treated 20 stroke patients aged 40–60, from 6 to 18 months after onset, with ankle plantar flexors spasticity. They concluded that fSWT was effective in controlling spasticity (H/M ratio), increase dorsiflexion active range of motion of the ankle and improving 10-m walking test in stroke patients compared to 20 controls treated with sham-fSWT.

Taheri et al. (2017) [52] studied the effect of fSWT on LL spasticity in 28 eligible stroke patients (22 ischemic, aged 18 to 70 years, divided into two groups. The first group (33 ± 21.4 months after onset) received one session per week for three weeks of ESWT, along with oral antispastic medications and stretching exercises. The control group (25.8 ± 9.9 months after onset) received only oral antispastic medications and stretching exercises similar to the first group. They concluded that fSWT combined with oral antispastic medications and stretching exercises significantly decreased LL spasticity in MAS, pain, passive range of motion, 3-m walk duration, and lower extremity functional score (LEFS) immediately and 12 weeks after treatment.

Yoon et al. (2017) [56] from Korea treated spastic elbow flexor and wrist pronator with rSWT in 21 patients (13 men, 12 ischemic) in the age of 57.4 ± 12.6 years from four to 24 months after stroke, one session/week, total 3 sessions in each. They observed significant improvement in muscle tone of elbow flexor and wrist pronator after four weeks compared with baseline and sham stimulation (*p* < 0.001). The active elevation of the hemiplegic upper limb (UL) was significantly increased (*p* < 0.05).

Wu et al. (2017) [53] performed the first and only one comparative study assessing the effects of rSWT and fSWT in post-stroke spasticity among 32 patients. Three ESWT sessions were administered to the triceps surae muscle within one-week interval (3000 pulses to gastrocnemius muscle and 1500 pulses to soles muscle with 2.0 bars, 0.1 mJ/mm^2^ and 5 Hz). Clinical assessments (MAS, MTS and ROM as well as dynamic foot plantar contact area and gait speed) were performed at one, four, and eight weeks. MAS and MTS were improved significantly in both rSWT and fESW groups. The remaining outcomes (except gait speed—insignificant changes) have been significantly improved in both groups, however greater after rSWT.

Lee et al. (2018) [54] randomly assigned 18 post-stroke patients between the ages of 30 and 70 years at least three months after onset to an fSWT group (*n* = 9) or control group (*n* = 9). In the first group, a single session of fSWT was given in the medial head of the gastrocnemius muscle of the spastic side at 4 Hz, 2000 shots with the EFD of 0.1 mJ/mm^2^. Sham stimulation was provided by only making sound without putting the device into contact with the skin in the control group. There were no significant differences between both groups. At all follow-up evaluations, the improvement was shown in MAS and changes from baseline of ultrasonographic measures in the fSWT group compared to the control group.

Radinmehr et al. (2019) [55] compared therapeutic ultrasound (US) and rSWT to treat plantar flexor spasticity after stroke. In a prospective, single-blind, randomized clinical trial, 32 patients (19 male, age range 42–78 years) with chronic stroke were randomly divided into two groups: the US group (*n* = 16) received the continuous ultrasound, intensity 1.5 w/cm^2^, frequency 1 MHz, and duration 10 min. The rSWT group (*n* = 16) was treated with rSWT, 0.340 mJ/mm^2^, 2000 shots. Both groups received the treatments for one session. The H-reflex tests of Hmax/Mmax ratio and H-reflex latency, the MAS), active range of motion (aROM), passive range of motion (pROM), passive plantar flexor torque (pPFT), and the timed “up and go” test (TUG) were blinded assessed at baseline (T0), immediately post-treatment (T1), and one-hour follow-up (T2). The result: the MMAS spasticity scores, aROM and pROM, pPFT, and TUG improved significantly within groups. The H-reflex tests did not improve across the groups. The results found no significant differences between groups for all outcome measures.

### 3.3. Recent Reviews and Meta-Analyses

Lee et al. (2015) [58] made a meta-analysis of the effects of fSWT on spasticity in patients after brain lesions. Five studies were ultimately included in the meta-analysis (27 subjects with cerebral palsy and 60 with stroke). The result: the MAS grade was significantly improved immediately after fSWT compared with the baseline values (standardized mean difference (SMD), −0.792; 95% confidence interval (CI), −1.001 to −0.583). The MAS grade at four weeks after ESWT was also significantly improved compared with the baseline values (SMD, −0.735; 95% CI, −0.951 to −0.519). In conclusion: ESWT has a significant effect on improving spasticity.

Dymarek et al. (2016) [23] published the results of the narrative review on the effects of rSWT on UL and LL spasticity in post-stroke patients. Ultimately, eight clinical studies within a total of 195 patients met the inclusion criteria for this review. Only one randomized controlled trial was found and then scored using the Cochrane-based assessment. The other studies presented low methodological quality. In conclusion: ESWT was found to be safe and effective. The mechanism of ESW action is still under investigation.

Guo et al. (2017) [59] confirmed the positive effects of ESWT on spasticity in post-stroke patients. Six studies consisting of nine groups, with a total of 160 patients, were included in this meta-analysis. The MAS grades immediately after ESWT were significantly improved compared with the baseline values.

Xiang et al. (2018) [60] published their results of systematic review and meta-analysis of randomized controlled trials on the effects of ESWT on spasticity in post-stroke patients. Eight randomized controlled trial studies (*n* = 385 patients) met the inclusion criteria. There was a high level of evidence that ESWT significantly ameliorates spasticity in post-stroke patients according to the four parameters: MAS, MTS, H/M ratio (maximum H reflex to maximum M response), and range of motion. However, there was no statically significant difference on the MAS at four weeks.

Oh et al. (2019) [61] published results of a meta-analysis on the duration of treatment effect of ESWT on spasticity and according to the number of shocks and application site. Total of nine trials met the inclusion criteria. There were 285 patients representing three groups: stroke, multiple sclerosis, and cerebral palsy. Patients after stroke (*n* = 152) were treated with ESWT one to 198 month after onset. In studies included in this paper, ESWT was performed 1500 to 18,000 times with an intensity of 0.03 mJ/mm^2^. The estimated effect size showed statistically significant MAS grade reduction immediately after treatment one week after, four weeks after, and 12 weeks after treatment. The number of pulses or site of application (knee and ankle joints vs. elbow, wrist, and finger joints) had no significant influence on the therapeutic effect of ESWT in reducing spasticity.

The most current metanalyses of the same authors Cabanas-Valdés et al. [62,63] have been published separately for the LL (2020) [62] and UL (2020) [63]. The first one selected total of 12 studies (5 RCTs, 1 CCTs, and 6 PCTs) and finally analyzed 5 RCT studies among 278 post-stroke patients. The authors concluded that both types of ESWT are non-invasive and effective in to reducing lower limb spasticity, increasing ankle ROM, and improving lower limb function in chronic stroke patients. What is more, ESWT does not show any side effects and can be consider as safe and effective method [62]. In turn the second metanalysis on UL muscles qualified 24 articles for qualitative synthesis and 16 articles for metanalysis. The authors provided a valuable summary of their analysed population including 764 patients (262 female and 502 male) with a mean age from 47 to 69 years old, of which 412 had ischemic and 280 had hemorrhagic stroke as well as 161 patients had left-sided and 200 had right-sided hemiparesis. It was found that ESWT is an effective non-invasive modality for clinical practice aimed at reduction of the upper limb muscles’ spasticity after stroke. The more favorable effects were found where ESWT was combined with conventional rehabilitation programs. It should be emphasized that the effect ESWT on motor function and recovery after stroke is limited [63].

Furthermore, Jia et al. (2020) [30] assessed the effects of ESWT on post-stroke spasticity in the long-term perspective. The study outcomes were the MAS, VAS, ROM, and FMA as well as adverse events were observed. A total of 8 RTCs have been extracted including 301 patients. The long-term follow-up finings revealed that ESWT significantly reduced MAS (95% CI = 0.53 to 0.19, I2 = 68%; *p* < 0.001) and VAS (95% CI = 1.51 to 0.37, I2 = 15%; *p* = 0.001), increased ROM (95% CI = 2.76 to 9.18, I2 = 0%; *p* < 0.001) and improved FMA (95% CI = 0.29 to 2.24, I2 = 96%; *p* = 0.01). The authors emphasized that optimal ESWT parameters, such as intensity, frequency, and number of pulses need to be explored in future studies.

## 4. Discussion

There are three main goals of spasticity treatment: to improve function, reduce the risk of unnecessary complications and relieve pain. Although the mechanism of the therapeutic effects of ESWT is still unknown, the majority of published papers have shown positive and beneficial effects of using ESWT as a treatment for musculoskeletal disorders, while the complications are low or negligible. So far, the specific mechanism of the antispastic action of ESWT is still unknown. In the presented above reports, ESWT started to be used in one month after stroke onset and, on the other hand, 198 months after onset. ESWT was applied one time, two times or three times in a one-week interval. The authors applied a different number of pulses (shots): from 1200 till 18,000, intensity differed between 0.03 and 1.95 mJ/mm^2^ (1.5 to 4 bar), and frequency from 4 to 12 Hz. There is still open the question: where should be ESWT applied: to the muscle belly, the distal or proximal muscle attachment?

Various controls have been used. In a small number of studies, firstly shame—shock wave treatment has been used before ESWT. In some studies, the patients have been treated parallel pharmacologically. The control group usually has been constructed with patients treated with the botulinum toxin, electrostimulation, or ultrasounds [1,24,36,42,64,65]. Table 7 presents implications for clinical practice and methodological issues when using ESWT for post-stroke spasticity management.

Different outcome measures have been used. As for primary outcome measure of spasticity most frequently has been used Ashworth scale and [66], as well as MTS [67], spasm frequency. As for secondary outcome measures usually aROM and pROM, ADL—mostly Barthel Index (BI), modified Barthel Index (MBI), MRS, hand/arm function—mostly UL-FMA, Timed Up and Go test (TUG), and Visual-Analogue Scale (VAS) has been used. In some studies, the neurophysiologic evaluation has been done (F wave, H-reflex, and H/M ratio) [29].

It is worth stressing that out of 19 reports, only three studies in fSWT and four in rSWT obtained Sackett’s grading system’s highest Level 1 of evidence [68]. A methodological question arises: What is the “ideal” research design for assessing the best parameters of ESWT in post-stroke spasticity? This should be RCT with large groups of patients and precise inclusion criteria.

The enrolled patients should be after first-ever stroke, ischemic, or hemorrhagic (except subarachnoid) with hemiparesis. The question is: Should we exclude quadriplegic patients and/or suffering from hemianesthesia? As for age: Should we limit the age to (e.g.,) 80 years because of comorbidity, which could change the muscle tone? We should give an essential question: Is it worth to treat spasticity later than (i.e.,) five years (60 months) after stroke onset? One must consider that after stroke many structural, irreparable changes occur, like muscle atrophy, loss of sarcomeres, abnormal actin and myosin cross-bridges, thixotropy, connective tissue remodeling, intra-articular adhesion formation, etc. Which level of spasticity, according to the Ashworth scale or MAS would be optimal? In our opinion, the best criterion would be from 2 to 3. Is it not worth to treat patients with spasticity of level 1+ because this level is right for the patient (better blood circulation and joint stabilization).

It is obvious that any pharmacological antispastic treatment (or any that could influence muscle tone) should be withdrawn at least two weeks before the experiment. Comprehensive rehabilitation should be continued, but without any muscle’s stimulation. Which would be the best control group? We recommend sham-ESWT. As for the number of sessions: one dose seems to be too little, optimal should be three sessions in a one-week interval. Should we use fSWT or rSWT? One should take into consideration that the rSWT is deemed to be less invasive than fSWT, and the rSWT devices are less expensive. Which should be the best parameters of ESWT: pressure—should be checked (i.e.,) between 1.0 and 2.0 bar; energy flux density 0.1 vs. 0.5 mJ/mm^2^, number of pulses: should be checked 1500 vs. 4000, frequency—should be checked 4 vs. 8 Hz. The most difficult question is: Which type of device should be recommended: electromagnetic, electrohydraulic, piezoelectric, or pneumatic?

As for the ESWT application site, it should be tried to compare the muscle belly vs. musculotendinous junction. As for the outcome measure: primary: Ashworth Scale (AS), MAS or MMAS [69,70]; secondary: pROM; body function: UL-FMA and 10-m time walk test (10-MWT); activity: Berg Balance Scale (BBS) and TUG; ADL: Barthel Index (BI) and FIM; and participation: Stroke Impact Scale (SIS) [71]. When should the outcome be measured? The optimal seems to be seven days after the last intervention, one-month, three-month, six-month, and 12-month follow-up. Table 8 presents research questions and suggestions for methodological improvements for future research.

## 5. Summary

This narrative review clearly reveals that the application of ESWT effectively reduces muscle tone in people with spastic limb after stroke. ESWT procedures are safe and free of undesirable side effects. The mechanism of action of ESWT on muscles affected by spasticity is still unknown. To date, no standard parameters of ESWT in post-stroke spasticity regarding intensity, frequency, location, and a number of sessions have been established. Further research, meeting the highest standards, is needed to establish recommended muscle stimulation parameters of using ESWT against spasticity.

## Figures and Tables

**Table 1:** Upper limb—characteristics of patients and outcomes.

Authors	Year	Sample (N)	Age (y)	Gender (M/F)	Duration (mo)	Stroke (I/H)	Outcomes	Side Effects
Manganotti and Amelio [35]	2005	20	63.0	11/9	>9	15/5	MAS (+), ROM (+), EMG (−)	not specified
Santamato et al. [36]	2013	16	64.4	9/7	10.5	8/8	MAS (+), SFS (+), VAS (+)	none
Troncati et al. [37]	2013	12	48.0	1/11	not specified	6/6	MAS (+), FMA (+), ROM (+)	not specified
Daliri et al. [38]	2015	15	54.4	12/3	≥6	13/2	MMAS (+), BRS (−), EMG (+)	not specified
Dymarek et al. [39]	2016	30	61.4	11/19	26–77	30/0	MAS (+), sEMG (+), IRT (+)	none
Dymarek et al. [40]	2016	20	63.1	13/7	9–120	20/0	MAS (+), sEMG (+), IRT (+)	none
Li et al. [41]	2016	20	55.4	12/8	9–144	10/10	MAS (+), FMA (+)	none
Kim et al. [44]	2016	17	59.9	7/10	4–60	8/9	VAS (+), CMS (+), ROM (−), FMA (−), MAS (−)	petechiae, bulla
Yoon et al. [56]	2017	26	58.7	26/0	2–198	not specified	MAS (+), MTS (+)	not specified
Yoon et al. [56]	2017	28	63.1	27/1	2–198	not specified	MAS (+), MTS (+)	not specified
Wu et al. [42]	2018	21	60.0	8/13	>60	11/9	MAS (+), ROM (+), FMA (+)	none
Park et al. [43]	2018	15	64.2	9/6	8.1	10/5	FMA (+), STM (+)	not specified
Li et al. [44]	2020	27	65.0	20/7	>1	24/3	MAS (+), VAS (+), MTS (+), FMA (−)	not specified

Legend: N, number of participants; y, years old; M, male; F, female; mo, months; I, ischemic; H, hemorrhagic; MAS, Modified Ashworth Scale; MMAS, Modified Modified Ashworth Scale; ROM, Range Of Motion; EMG, electromyography; SFS, Spasm Frequency Scale; VAS, Visual-Analogue Scale; FMA, Fugl–Meyer Assessment; BRS, Brunnstrom Recovery Stage; sEMG, surface electromyography; IRT, infrared thermography; MTS, Modified Tardieu Scale; STM, soft tissue myotonometry; (+), substantially improved outcome; (−), substantially unchanged outcome.

**Table 2:** Upper limb—characteristics of SWT procedure and treatments.

Authors	Year	Sessions [N]	Pulses [N]	F [Hz]	P [bars]	EFD [mJ/mm^2^]	Active-SWT	Sham-SWT	Local Anesthesia	Additional Therapy
Manganotti and Amelio [35]	2005	1	1500/3200	not specified	1.5	0.03	fSWT	none	none	not specified
Santamato et al. [36]	2013	5	2000	4	1.5	0.1	fSWT	none	none	not specified
Troncati et al. [37]	2013	2	1600/3200	not specified	not specified	0.08	fSWT	none	not specified	not specified
Daliri et al. [38]	2015	1	1500	4	1.5	0.03	rSWT	sound, without energy	not specified	not specified
Dymarek et al. [39]	2016	1	1500	4	1.5	0.03	rSWT	plastic cover	none	none
Dymarek et al. [40]	2016	1	1500	4	1.5	0.03	rSWT	plastic cover	none	none
Li et al. [41]	2016	3	2750	4	3.3	0.2	rSWT	none	none	rehabilitation
Kim et al. [44]	2016	3	1500	12	2.0	0.1	rSWT	without trans-mitter	none	not specified
Yoon et al. [56]	2017	3	1500	5	1.5	0.08	fSWT	sound, non-contact	not specified	antispastic drugs and physiotherapy
Wu et al. [42]	2018	3	3000	5	3.5	0.2	fSWT	none	none	activity training
Park et al. [43]	2018	16	1500/3200	not specified	1.5	0.03	fSWT	sound, without energy	not specified	not specified
Li et al. [44]	2020	5	6000	18	1.2–1.4	0.06–0.07	rSWT	none	none	physical therapy

Legend: N, number; F, frequency; P, pressure; EFD, energy flux density; SWT, shock wave therapy; fSWT, focused shock wave therapy; rSWT, radial shock wave therapy.

**Table 3:** Upper limb—methodological quality and level of evidence of studies.

Authors	Year	Journal	Protocol	1. Eligibility Criteria *	2. Random Allocation	3. Concealed Allocation	4. Baseline Comparability	5. Blind Subjects	6. Blind Therapists	7. Blind Assessors	8. Adequate Follow-Up	9. Intention-To-Treat Analysis	10. Between-Group Comparisons	11. Point and Variability Measures	Total Score in PEDro	Evidence Level in NICE	Level of Recommendation in NICE
Manganotti and Amelio [35]	2005	Stroke	CCT	+	−	−	+	−	−	−	+	−	+	+	4	2++	B
Santamato et al. [36]	2013	Ultrasound in Medicine and Biology	RCT	+	+	−	+	−	−	+	+	−	+	+	6	1+	
Troncati et al. [37]	2013	NeuroRehabilitation	CCS	+	−	−	−	−	−	−	+	−	−	+	2	2−	
Daliri et al. [38]	2015	NeuroRehabilitation	RCT	+	−	−	+	+	−	−	−	−	+	+	4	1−	
Dymarek et al. [39]	2016	Ultrasound in Medicine and Biology	RCT	+	+	−	+	+	−	−	+	−	+	+	6	1+	
Dymarek et al. [40]	2016	Evidence-Based Complementary and Alternative Medicine	PCT	+	−	−	−	−	−	−	+	−	−	+	2	2−	
Li et al. [41]	2016	Medicine (Baltimore)	RCT	+	+	−	+	+	−	+	+	+	+	+	8	1++	
Kim et al. [44]	2016	Annals of Rehabilitation Medicine	RCT	+	+	−	+	+	−	+	+	−	+	+	7	1+	
Yoon et al. [56]	2017	Annals of Rehabilitation Medicine	RCT	+	+	−	+	−	−	−	+	−	+	+	5	1−	
Wu et al. [42]	2018	Archives of Physical Medicine and Rehabilitation	RCT	+	+	+	+	−	+	+	+	−	+	+	8	1++	
Park et al. [43]	2018	Journal of Physical Therapy Science	RCT	+	+	+	−	+	−	−	−	−	+	+	6	1+	
Li et al. [44]	2018	Age and Ageing	RCT	+	+	+	+	−	−	+	−	+	+	+	7	1+	

Notes: * criterion 1 does not contribute to the total PEDro score. Legend: PEDro, Physiotherapy Evidence Database; NICE, National Institute for Health and Clinical Excellence; RCT, randomized controlled trial; CCT, clinical controlled trial; CCS, clinical case series; PCT, prospective clinical trial.

**Table 4:** Lower limb—characteristics of patients and outcomes.

Authors	Year	Sample [N]	Age [y]	Gender [M/F]	Duration [mo]	Stroke [I/H]	Outcomes	Side Effects
Sohn et al. [46]	2011	10	44.9	6/4	23–77	2/8	MAS (+), EMG (−)	not specified
Moon et al. [47]	2013	30	52.6	17/13	80.5	16/14	MAS (+), ROM (−), FMA (−), IDT (+)	none
Santamato et al. [48]	2014	23	51.6	15/8	24.9	12/11	MAS (+), ROM (+), EMG (−)	pain, weakeness
Kim et al. [49]	2015	10	64.1	5/5	17.6	5/5	STM (+), VAS (+), VGA (+)	not specified
Radinmehr et al. [50]	2017	12	59.0	7/5	34.0	6 /6	MMAS (+), ROM (+), IKD (+), TUG (+), EMG (−)	none
Sawan et al. [51]	2017	20	50.6	9/4	6–18	not specified	EMG (+), ROM (+), 10-mWT (+)	not specified
Taheri et al. [52]	2017	14	44.0	9/4	12–55	11/2	MAS (+), VAS (+), ROM (+), 3-mWT (+), LEFS (+)	not specified
Yoon et al. [56]	2017	13	61.0	13/0	12–184	not specified	MAS (+), MTS (+)	not specified
Yoon et al. [56]	2017	13	66.9	13/0	15–87	not specified	MAS (+), MTS (+)	not specified
Wu et al. [53]	2018	31	59.9	18 /13	50–55	20/11	MAS (+), MTS (+), ROM (+), 10-MWT (+), FPMP (+)	none
Lee et al. [54]	2018	9	50.0	7/2	>3	4/5	MAS (+), ROM (+), FMA (+), USG (+)	not specified
Radinmehr et al. [50]	2019	16	60.0	9/7	>1	not specified	MMAS (+), ROM (+), IKD (+), TUG (+), EMG (−)	none

Legend: N, number of participants; y, years old; M, male; F, female; mo, months; I, ischemic; H, hemorrhagic; MAS, Modified Ashworth Scale; EMG, electromyography; FMA, Fugl–Meyer Assessment; ROM, Range Of Motion; VAS, Visual-Analogue Scale; CMS, Constant-Murley Score; MMAS, Modified Modified Ashworth Scale; IKD, isokinetic dynamometry; TUG, Timed Up and Go; 10-mWT, 10-m Walk Test; 3-mWT, 3-m Walk Test; LEFS, Lower Extremity Functional Scale; MTS, Modified Tardieu Scale; USG, ultrasonography; STM, soft tissue myotonometry; (+), substantially improved outcome; (−), substantially unchanged outcome.

**Table 5:** Lower limb—characteristics of SWT procedure and treatments.

Authors	Year	Sessions [N]	Pulses [N]	F [Hz]	P [bars]	EFD [mJ/mm^2^]	Active-SWT	Sham-SWT	Local Anesthesia	Additional Therapy
Sohn et al. [46]	2011	1	1500	not specified	2.0	0.15	fSWT	none	none	antispastic drugs and physiotherapy
Moon et al. [47]	2013	3	1500	4	2.0	0.09	fSWT	none	none	rehabilitation
Santamato et al. [48]	2014	1	1500	not specified	1.5	0.03	fSWT	none	none	none
Kim et al. [49]	2015	3	1500	4	1.5	0.089	not specified	none	none	rehabilitation
Radinmehr et al. [50]	2017	1	2000	5	3.0	0.3	rSWT	none	not specified	none
Sawan et al. [51]	2017	1	1500	not specified	not specified	not specified	fSWT	none	not specified	physical therapy
Taheri et al. [52]	2017	3	1500	4	1.5	0.1	fSWT	sound, without energy	not specified	antispastic drugs and physiotherapy
Yoon et al. [56]	2017	3	1500	5	1.5	0.08	fSWT	sound, without contact	not specified	antispastic drugs and physiotherapy
Wu et al. [53]	2018	3	3000	5	2.0	0.1	fSWT rSWT	none	not specified	not specified
Lee et al. [54]	2018	1	2000	4	1.5	0.08	fSWT	sound, without contact	not specified	physical therapy
Radinmehr et al. [50]	2019	1	2000	5	1.5	0.08	fSWT	none	not specified	physical therapy

Legend: N, number; F, frequency; P, pressure; EFD, energy flux density; SWT, shock wave therapy; fSWT, focused shock wave therapy; rSWT, radial shock wave therapy.

**Table 6:** Lower limb—methodological quality and level of evidence of studies.

Authors	Year	Journal	Protocol	1. Eligibility Criteria *	2. Random Allocation	3. Concealed Allocation	4. Baseline Comparability	5. Blind Subjects	6. Blind Therapists	7. Blind Assessors	8. Adequate Follow-Up	9. Intention-To-Treat Analysis	10. Between-Group Comparisons	11. Point and Variability Measures	Total Score in PEDro	Evidence Level in NICE	Level of Recommendation in NICE
Sohn et al. [46]	2011	Annals of Rehabilitation Medicine	CCT	+	−	−	−	−	−	−	+	−	−	+	2	2−	C
Moon et al. [47]	2013	Annals of Rehabilitation Medicine	CCT	+	−	−	+	−	−	−	+	−	+	+	4	2++	
Santamato et al. [48]	2014	Topics in Stroke Rehabilitation	PCT	+	−	−	−	−	−	−	−	−	−	+	1	2−	
Kim et al. [48]	2015	Journal of Physical Therapy Science	PCT	+	−	−	−	−	−	−	+	−	−	+	3	2+	
Radinmehr et al. [49]	2017	Disability and Rehabilitation	RCT	+	+	−	−	+	+	−	−	−	+	+	5	1−	
Sawan et al. [50]	2017	NeuroRehabilitation	CCT	+	−	−	−	−	−	−	+	−	+	+	3	2+	
Taheri et al. [51]	2017	Archives of Iranian Medicine	RCT	+	+	−	+	−	−	−	+	−	+	+	5	1−	
Yoon et al. [56]	2017	Annals of Rehabilitation Medicine	RCT	+	+	−	+	−	−	−	+	−	+	+	5	1−	
Wu et al. [53]	2018	European Journal of Physical and Rehabilitation Medicine	RCT	+	+	+	+	+	+	−	+	−	+	+	8	1++	
Lee et al. [54]	2018	Physical Medicine and Rehabilitation	RCT	+	+	+	+	+	+	+	+	−	+	+	9	1++	
Radinmehr et al. [50]	2019	Journal of Stroke and Cerebrovascular Diseases	RCT	+	+	+	+	−	−	+	−	+	+	+	7	1+	

Notes: * criterion 1 does not contribute to the total PEDro score. Legend: PEDro, Physiotherapy Evidence Database; NICE, National Institute for Health and Clinical Excellence; RCT, randomized controlled trial; CCT, clinical controlled trial; CCS, clinical case series; PCT, prospective clinical trial.

**Table 7:** Implications for clinical practice in using ESWT for post-stroke spasticity management.

Practical Implications	
Practical Component	Explanation and Justification
fSWT devices	EM, EH, or PE devices: therapeutic effects on the level of cells (molecular changes)
rSWT devises	PN devices: therapeutic effects on the level of tissues (morphological changes)
UL muscles	Biceps brachii, flexor carpi radialis, flexor carpi ulnaris, palmar interosseous, flexor digitorum superficialis
LL muscles	Triceps surae, biceps femoris
P [bar]	Typically, 1.5–2.0 but sometimes higher values even up to 3.0–3.5
EFD [mJ/mm^2^]	Typically, 0.03–0.1 but sometimes higher values even up to 0.2–0.3
TED [J/mm^2^] *	Typically, 0.05–0.45 but sometimes higher values even up to 0.9–1.2
F [Hz]	Normally, range of 4–5 but some reports indicate even up to 12–18
Pulses [N]	Normally, 1500–2000 per muscle belly, occasionally to 3000, but also even up to 6000
Sessions [N]	Usually, 1–2 per week and 3–5 totally during treatment period, sometimes even up to 16
ESWT transducer	Applicator head placed perpendiculary and directly over the muscle belly or myotendinous junction
USG gel application	Ultrasonic gel as a contact medium applied on the skin within the treatment area to reduce tissue resistance
LA administration	Usually not recommended but it depends on individual pain threshold of each patients
AE risk management	Should be carefully observed and reported such as local episodes: pain, bruises, petechiae, muscle weakness

Notes: * Total energy density is calculated as the product of the number of sessions, the number of pulses, and the energy flux density. Legend: ESWT, extracorporeal shock wave therapy; fSWT, focused shock wave therapy; EM, electromagnetic; EH, electrohydraulic; PE, piezoelectric; rSWT, radial shock wave therapy; PN, pneumatic; UL, upper limb; LL, lower limb; P, pressure; EFD, energy flux density; TED, total energy density; F, frequency; N, number; USG, ultrasonic gel; LA, local anesthesia; AE, adverse events.

**Table 8:** Research questions and suggestions for future research directions.

Future Directions	
Research Questions	Methodological Advices and Research Directions
1. How to improve the research methodology?	Multicenter, placebo controlled RCT should be considered with better quality according to PEDro, NICE tools (see Table 3 and Table 6) but also Cochrane Collaboration guidelines [72,73] *. So far, only a few studies met the highest methodological quality with a PEDro score between 7 and 9, there is no study with PEDro score 10.
2. Which patients should be enrolled in the study?	It is important to qualify a homogeneous group of patients with precise consideration of the baseline characteristics such as stroke etiology, duration of stroke, functional ability level (e.g., IB), spasticity grade (e.g., MAS). From the scientific perspective it seems interesting to divide patients according to the duration of the stroke in order not to compare short-term spasticity with permanent spasticity; it is also useful to divide patients according to MAS grade to check if clinical effects are comparable in patients with low spasticity (MAS 1/1+) to those with persistent spasticity (MAS 3/4). Further, comorbidities directly excluding the patients (e.g., SAH) and potentially disturbing the proper course of the study as well as any potential contraindications for ESWT should be carefully checked.
3. Which study outcomes should be considered?	The verification of study outcomes should use both subjective methods of assessment based on questionnaires and scales as well as most importantly objective research methods with the use of research equipment to provide measurable results and repeatable measurements (see Table 2 and Table 4). Both short-term effects (immediate, e.g., 12, 24, and 48 h) and long-term effects (follow-up, e.g., 1, 4, and 8 weeks or even 6 and 12 months) assessments should be performed to extend the observation spectrum. Despite the proven safety of ESWT in stroke patients, most studies neglect to observe and report potential ESWT-related adverse events.
4. How should the shock wave treatment be performed?	ESWT, both rSWT and fSWT, should be applied directly to the spastic muscles, however, the treatment area should include both muscle bellies and musculotendinous junctions to make the procedure effective. Different treatment parameters such as number of pulses, energy and frequency should be applied, compared, and verified under the same clinical conditions to identify the most effective combinations of these parameters. So far, there is only one clinical study by Wu et al. (2018) [53] which directly compares the clinical effectiveness of rSWT and fSWT. It is also important to determine the optimal position for the patient during treatment sessions according to the level of functional ability.
5. How to perform the sham procedure in placebo group?	Placebo procedures in ESWT studies are performed as sham-ESWT (passive interventions) and different methods are used for this purpose. There is the possibility to use only the characteristic sound of shocks generated by the ESWT without energy delivery to the tissues (inactive applicator) or with minimal energy without therapeutic effect. It is possible to carry out sham-ESWT treatments using a non-contact method or with the applicator head covered by a polyethylene energy-absorbing cap. Some researchers use transmitters specially adapted by manufacturers to carry out studies with placebo intervention. Other researchers remove the ESWT transmitter on their own to exclude the transmission of energy to the patient’s tissues.

Notes: * Cochrane Collaboration’s tool for assessing risk of bias in randomized clinical trials by Higgins and Altman. Legend: RCT, randomized controlled trial; PEDro, Physiotherapy Evidence Database; NICE, National Institute for Health and Clinical Excellence; ESWT, extracorporeal shock wave therapy; rSWT, radial shock wave therapy; fSWT, focused shock wave therapy; SAH, subarachnoid hemorrhage; IB, Index Barthel; MAS, Modified Ashworth Scale.

## Data Availability

The data presented in this study are available in the article.

## Abbreviations

ADL	Activities of daily living
aROM	active range of motion
BI	Barthel Index
BTX-A	botulinum toxin type A
CI	confidence interval
CIMT	constraint-induced movement therapy
CNS	central nervous system
EFD	energy flux density
EMG-BF	electromyographic biofeedback training
EMG-NMES	electromyography neuromuscular electrical stimulation
ES	electrical stimulation
ESWT	extracorporeal shock wave therapy
FES	functional electrical stimulation
FGF	fibroblast growth factors
fSWT	focused shock wave therapy
IRT	infrared thermography
LBP	low back pain
LL	lower limb
MAS	Modified Ashworth Scale
MBI	Modified Barthel Index
MMAS	Modified Modified Ashworth scale
MRS	Modified Rankin Scale
MS	multiple sclerosis
MTS	Modified Tardieu Scale
MVFT	mirror visual feedback training
NCS	neural stem cell
NDT	neurodevelopmental treatment by Bobath
NICE	National Institute for Health and Clinical Excellence
NMES	neuromuscular electrical stimulation
NO	nitric oxide
NTH-3	neurotrophin-3
PEDro	Physiotherapy Evidence Database
PNF	proprioceptive neuromuscular facilitation
pPFT	plantar flexor torque
pSWT	planar shock wave therapy
RAT	robot-assisted training
rSWT	radial shock wave therapy
rTMS	transcranial magnetic stimulation
SCI	spinal cord injuries
sEMG	surface electromyography
SMD	standardized mean difference
SMT	sensimotor movement training
SP	substance P
SS	swelling scale
tDCS	transcranial direct current stimulation
TENS	transcutaneous electrical nerve stimulation
TGF	transforming growth factor
TRT	task-related training
TU	therapeutic ultrasound
UL	upper-limb
UL-FMA	Upper Limb—Fugl–Meyer Assessment
UMN	upper motor neuron
VAS	Visual-Analogue Scale
VEGF	vascular endothelial growth factor
VRBT	virtual reality-based training.
WBVT	whole body vibration training

## Appendix A

**Table A1:** Methodological quality of studies according to PEDro score [33].

PEDro Score	
Criteria	Explanation
1. *	Eligibility criteria were specified.
2.	Subjects were randomly allocated to groups.
3.	Allocation was concealed.
4.	The groups were similar at baseline regarding the most important prognostic indicators.
5.	There was blinding of all subjects.
6.	There was blinding of all therapists who administered the therapy.
7.	There was blinding of all assessors who measured at least one key outcome.
8.	Measures of at least one key outcome were obtained from more than 85% of the subjects initially allocated to groups.
9.	All subjects for whom outcome measures were available received the treatment or control condition as allocated or, where this was not the case, data for at least one key outcome was analysed by “intention to treat”.
10.	The results of between-group statistical comparisons are reported for at least one key outcome.
11.	The study provides both point measures and measures of variability for at least one key outcome.

Notes: * criterion 1 does not contribute to the total score. Legend: PEDro, Physiotherapy Evidence Database.

**Table A2:** Levels of evidence for intervention studies according to NICE guidelines [34].

NICE Guidelines	
Level of Evidence	Type of Evidence
1++	High-quality meta-analyses, systematic reviews of RCTs, or RCTs with a very low risk of bias.
1+	Well-conducted meta-analyses, systematic reviews of RCTs, or RCTs with a low risk of bias.
1−	* Meta-analyses, systematic reviews of RCTs, or RCTs with a high risk of bias. *
2++	High-quality systematic reviews of case-control or cohort studies. High-quality case-control or cohort studies with a very low risk of confounding, bias or chance, and a high probability that the relationship is causal.
2+	Well-conducted case-control or cohort studies with a low risk of confounding, bias or chance, and a moderate probability that the relationship is causal.
2−	Case control or cohort studies with a high risk of confounding, bias or chance, and asignificant risk that the relationship is not causal. *
3	Nonanalytic studies (e.g., case reports and case series).
4	Expert opinion, formal consensus.

Notes: * Studies with a level of evidence “1−” and “2−” are not used for developing clinical recommendations. Legend: NICE, National Institute for Health and Clinical Excellence; RCT, randomized controlled trial.

**Table A3:** Gradings of recommendations according to NICE guidelines [34].

NICE Guidelines	
Grade	Explanation
A	At least one meta-analysis, systematic review, or randomized controlled trial rated as 1++ and directly applicable to the target population or a systematic review of randomized controlled trials or a body of evidence consisting principally of studies rated as 1+ directly applicable to the target population and demonstrating overall consistency of results.
B	A body of evidence including studies rated as 2++ directly applicable to the target population and demonstrating overall consistency of results or extrapolated evidence from studies rated as 1++ or 1+.
C	A body of evidence including studies rated as * 1− or 2+ directly applicable to the target population and demonstrating overall consistency of results or extrapolated evidence from studies rated as 2++.
D	Evidence level * 2−, 3 or 4 or extrapolated evidence from studies rated as 2+.

Notes: * Studies with a level of evidence “1−” and “2−” are not used for developing clinical recommendations. Legend: NICE, National Institute for Health and Clinical Excellence; RCT, randomized controlled trial.
